# Attention is necessary for subliminal instrumental conditioning

**DOI:** 10.1038/srep12920

**Published:** 2015-08-10

**Authors:** Tommaso Mastropasqua, Massimo Turatto

**Affiliations:** 1 Center for Mind/Brain Sciences, University of Trento, Italy; 2 Department of Psychology and Cognitive Sciences, University of Trento, Italy

## Abstract

The capacity of humans and other animals to provide appropriate responses to stimuli anticipating motivationally significant events is exemplified by instrumental conditioning. Interestingly, in humans instrumental conditioning can occur also for subliminal outcome-predicting stimuli. However, it remains unclear whether attention is necessary for subliminal instrumental conditioning to take place. In two experiments, human participants had to learn to collect rewards (monetary gains) while avoiding punishments (monetary losses), on the basis of subliminal outcome-predicting cues. We found that instrumental conditioning can proceed subconsciously only if spatial attention is aligned with the subliminal cue. Conversely, if spatial attention is briefly diverted from the subliminal cue, then instrumental conditioning is blocked. In humans, attention but not awareness is therefore mandatory for instrumental conditioning, thus revealing a dissociation between awareness and attention in the control of motivated behavior.

Instrumental conditioning^1^,^2^ is a form of biological learning that exemplifies a general and fundamental principle governing human and animal behavior: actions followed by pleasant outcomes are likely to be repeated, whereas those followed by unpleasant outcomes tend to decrease over time^3^. This principle relies on the natural tendency of an organism to normally seek rewards and avoid punishments, and the primary reason for this behavioral bias is that rewards (e.g., food) tend to increase the fitness of the organism, whereas punishments may be dangerous for its survival. Instrumental conditioning thus represents a fundamental type of associative learning that allows the organism to exert behavioral control over motivationally significant events in an ever-changing environment^4^.

Learning which environmental stimuli anticipate the occurrence of rewards or punishments facilitates the selection of appropriate behavioral responses. For example, as the hungry pigeon in the Skinner box may learn that pecking the red disk leads to a food reward, humans may learn that the “On Sale!” sign indicates that they can buy valuable goods at affordable prices. Therefore, outcome-predicting cues are salient events we are usually well aware of. However, a recent study has shown that instrumental conditioning can take place even when humans are unaware of the reward/punishment cues^5^; also see^6^. This finding challenges the quite intuitive idea that cues anticipating motivationally significant outcomes are consciously represented in the human mind. Conversely, it shows that our actions can be unconsciously driven by subliminal reward/punishment cues, a result in agreement with other studies showing unconscious Pavlovian conditioning^7^.

Traditionally, unconscious processes have been considered to be automatic and independent of attention^8^,^9^, and more recent evidence suggests that attention and awareness can be distinct cognitive functions^10^. In light of this scenario, the case of subliminal instrumental conditioning raises the interesting question of whether this form of associative learning is also independent of attention, or, alternatively, whether subliminal outcome-predicting cues still require attentional processing for conditioning to occur.

The following two experiments were therefore aimed at addressing this question. To begin with, in Experiment 1 we wanted to confirm that instrumental conditioning can occur subliminally, driven by monetary gains and losses^5^. Hence, we presented a subliminal cue consisting of a masked arrow, which was paired either with a monetary win or a monetary loss, depending on its direction. After the arrow cue presentation participants had to decide whether or not they wanted to take the risk of accepting the following outcome (Fig. 1, top row; also see Methods for details). Furthermore, we wanted to provide further evidence that subliminal instrumental conditioning does not depend on the type of masking technique adopted to make the outcome-predicting cues invisible. To this aim, here we used a backward metacontrast masking^11^ in place of the forward-backward pattern masking^5^ or masking by crowding^6^ used before. Then, after having established that our metacontrast masking procedure was adequate to replicate the results reported previously^5^, in Experiment 2 we addressed the role of attention in subliminal instrumental conditioning. To this aim, we briefly diverted attention from the cue by means of a sudden onset presented in the opposite cue location (Fig. 1, bottom row; also see Methods for details). If spatial attention to the cue is necessary for subliminal instrumental conditioning, then participants should fail to learn the correct cue-outcome associations and their responses to reward and punishment cues should be indistinguishable. Alternatively, if spatial attention is not necessary, subliminal instrumental conditioning should emerge as in Experiment 1.

## Results

### Experiment 1

#### Cue visibility test

To ensure that the two cues presented during instrumental conditioning were subliminal, we used the same logic and procedure adopted by previous studies^5^. We calculated, for each of the participants, the *d’* value in the cue discrimination task performed after conditioning. Note that by testing the cue visibility after the conditioning phase we took a very conservative approach, as participants’ sensitivity to the cue could have improved because of training during the conditioning phase.

We assessed the statistical significance of *d’* using a bootstrapping procedure for each participant. In particular, we estimated the 95% confidence interval of *d’*, sampling with replacement from the observed data (sampling was repeated 10,000 times). By definition, a *d’* value was significantly different from zero, if the relative confidence interval did not contain zero. Four participants showed a *d’* > 0 (*mean d’* = 0.63), and were thus eliminated from the conditioning analysis reported below. None of the remaining 21 participants, when tested individually, were able to discriminate between the directions of the two cues (*mean d’* = 0.08).

#### Instrumental conditioning

To attest to the presence of subliminal instrumental conditioning we analyzed the proportion of “Yes” responses to the gambling task, namely the propensity of participants to accept the risk to win or lose money as a function of the subliminal cue. If subliminal instrumental conditioning were possible, then the proportion of “Yes” responses should be higher for the reward-predicting cue than for the punishment-predicting cue. The proportion of “Yes” responses were entered into a repeated measure ANOVA, with Cue type and Trial bin as factors (the 180 trials were divided into six 30-trial bins). The ANOVA showed a main effect of Cue type *F*(1, 20) = 13.416, *p* = 0.002, η^2^ = 0.401, and Trial bin *F*(5, 100) = 2.444, *p* = 0.039, η^2^ = 0.109, but not of their interaction. As depicted in Fig. 2a, the presence of subliminal instrumental conditioning was attested to by the fact that participants were more willing to accept the risk when presented with the subliminal cue paired with a monetary gain (reward), than with the cue paired with a monetary loss (punishment). The asymptotic value for the “Yes” responses in the reward and punishment functions was approximately 0.65 and 0.40 respectively, thus revealing a response pattern very similar to that obtained for the “go” responses in the previous study showing unconscious instrumental conditioning^5^.

To further substantiate the results depicted in Fig. 2a, the corresponding response pattern was also analyzed within the framework of Signal Detection Theory^12^, by treating “Yes” responses to rewards and punishments as *hits* and *false alarms* respectively, and by combining them into a single measure of sensitivity (*d’*) of the cue-outcome association. The analysis confirmed that participants’ sensitivity (*d’* = 0.449) in distinguishing between the two response alternatives as a function of the subliminal cue was larger than zero, *t*(20) = 3.337, *p* = 0.003 (also see Fig. 2c). Although Fig. 2a seems to suggest that learning was present already in the first bin of 30 trials, a more detailed analysis showed that learning was not significant in the first bin of 15 trials (see Fig. 3). Specifically, we observed a *d’* value of 0.216 (95% confidence interval = [−24; 456]).

It is worth noting that not all participants were able to learn the appropriate responses to the subliminal cues. Using the same bootstrapping procedure as in the cue visibility test, we found that 13 out of 21 participants (~62%) showed a *d’* greater than zero.

Another piece of evidence in support of the acquisition of conditioning was the amount of money won at the end of the task (€ 7.24), which was greater than that obtained when performance was at chance (€ 6), *t*(20) = 3.525, *p* = 0.002.

### Experiment 2

#### Cue visibility test

We calculated the *d’* value in the cue discrimination task performed after conditioning. The statistical significance of *d’* was calculated using the bootstrapping procedure described in Experiment 1. Three participants showed a *d’* > 0 (*mean d’* = 0.9), and were thus eliminated from the conditioning analyses reported below. None of the remaining 22 participants, when tested individually, were able to discriminate between the two cues directions (*mean d’* = 0.08).

#### Instrumental conditioning

We performed the same analyses conducted in Experiment 1. The ANOVA on the proportion of the “Yes” responses for the reward-predicting and punishment-predicting cues revealed that neither the main effect of Cue type *F*(1, 21) = 0.320, *p* = 0.577, η^2^ = 0.015, nor of Trial bin *F*(5, 105) = 0.370, *p* = 0.878, η^2^ = 0.017, were significant, and neither was their interaction. The lack of a significant Cue type effect showed that when spatial attention was shifted in the cue opposite location observers were not able to learn the relation between the subliminal cue and the paired outcome, and no instrumental conditioning was possible (see Fig. 2b). This was confirmed by the *d’* analysis, showing a lack of the participants’ sensitivity to the two response alternatives (*d’* = −0.018; *t*(21) = −0.504, *p* = 0.620; also see Fig. 2c). When analyzed individually, only 2 out of 22 participants (~9%) performed better than chance (*d’* > 0), whereas in Experiment 1 they were 13 out of 21 (~62%). In addition, a between-experiment comparison confirmed that *d’* was greater in Experiment 1 than in Experiment 2, *t*(41) = 3.421, *p* = 0.001 (Fig. 2c).

To further substantiate the learning difference between Experiment 1 and Experiment 2, in Fig. 4 we show the correlation across participants between *d*’ in the subliminal instrumental conditioning and *d*’ in the visibility test. As can be seen, the magnitude of conditioning was larger in Experiment 1 than in Experiment 2, as attested to by visual inspection of the intercepts of the two corresponding regression lines.

The absence of subliminal instrumental conditioning in Experiment 2 was also confirmed by the fact that the amount of money won in the task (€6) was identical to that obtained with a performance at chance (€6).

#### Spatial attention task

Response times (RTs) shorter or longer than 2 SD from the mean were trimmed before data analysis. This outlier-latency criterion removed less than 3% of the data. The results showed that participants were faster at responding on congruent (*M* = 502 ms) than on incongruent (*M* = 518 ms) trials, *t*(21) = 8.586, *p* < 0.001. This RTs pattern confirmed that the sudden onset of the oval stimulus induced a shift of spatial attention to the corresponding location.

## Discussion

Instrumental conditioning shows that individuals can learn from the consequences of their actions^1^,^2^,^3^. This fundamental form of associative learning requires a cognitive system capable of representing three events and their contingencies: a stimulus, a behavior, and an outcome^1^,^13^. Whether an individual needs to be aware of the (conditioned) stimulus predicting the trailing appetitive/aversive one is controversial in Pavlovian conditioning^14^. Yet instrumental conditioning does not involve the mere anticipation of a fixed reflex to the conditioned stimulus, but rather it requires learning to deliberately select the appropriate response as a function of the outcome-predicting cues^15^. Therefore, one would expect such cues to be consciously experienced during conditioning. This idea, however, has been challenged by recent studies documenting instrumental conditioning for subliminal cues^5^,^6^.

Here we have shown that, although subliminal instrumental conditioning is possible, still it depends on whether spatial attention is directed or not to the subliminal cues, a result in agreement with theories of associative learning in animals^16^. A natural complex environment may present several predictive relations between stimuli, but not all such relations are behaviorally relevant for the individual. Hence, an attentional driven conditioning mechanism seems to be an efficient evolutionary solution to restrict associative learning processes against those stimuli that predict motivationally significant outcomes, irrespective of awareness.

One may note that for half of the participants there was a spatial compatibility between cue direction and response key. For example, if the winning cue was the arrow pointing to the left when the left key indicated “Yes, I accept the risk”, then a direct motor specification mechanism could have explained the results of the training phase. However, we believe there are two reasons that seriously undermine this possibility. First, if it were true that for some participants there was a spatial compatibility between response key and cue direction, then the opposite would be true for the remaining participants. Therefore, if a direct motor specification had played a role in our paradigm, this should have produced two groups with opposite hit and false alarm rates functions, with the net result that the two opposite effects should have canceled each other out. Second, if during the training phase the direct motor specification had played a larger role in the spatial compatible group than in the other group, a direct motor specification mechanism would have led to a discriminative performance well above chance in the cue visibility test for all participants. This is because in the test phase there was always a perfect spatial compatibility between response key and cue direction. However, the results showed that this was not the case.

Although we interpreted our results as evidence that when attention was misaligned with the outcome-predicting cue learning was halted, an anonymous reviewer correctly noted that the lack of learning could have emerged because the shift of attention away from the cue increased the efficacy of masking, so that the processing of the cue was severely limited if not abolished. Indeed, previous studies have shown that attention can modulate the strength of metacontrast masking^17^. However, such modulation is ineffective when the interval between the target stimulus and the mask is similar to the one used in our study (40 ms). Attention modulates the masking power only at longer intervals (i.e., >80 ms)^17^. For this reason, it seems to us more likely that our attentional modulation affected the learning process directly, without altering the masking substantially. However, we acknowledge that the masking account cannot be ruled out completely.

Recent studies have shown that humans can learn subliminal and unattended visual features^18^,^19^, indicating that under certain conditions attention does not appear to be necessary for some types of learning. In contrast, here we documented that attention governs an associative learning process such as instrumental conditioning, even when the cues are subliminally perceived. The reasons why attention must be directed to the target feature in unconscious instrumental conditioning, whereas this seems to be unnecessary in task-irrelevant perceptual learning are not immediately clear. We can note, however, several differences between the two types of unconscious learning. First, unconscious (and conscious) instrumental conditioning requires the brain to learn an arbitrary stimulus-response mapping; by contrast, task-irrelevant perceptual learning involves changes in visual sensitivity, which is achieved through long-lasting modifications in the stimulus sensory representation. Second, while the former type of learning can take only a few trials to become manifest (depending on the complexity of the stimulus-response mapping), the latter requires thousands of trials to emerge. However, the mandatory role of attention for the processing of subliminal outcome-predicting stimuli is in accordance with a previous study showing that masked priming, though unconscious, still depends on attention being time-locked to the subliminal primer presentation^20^. Thus, our results provide further evidence in favor of a dissociation between awareness and attention in human cognition^10^.

## Methods

### Participants

Fifty undergraduate students (25 in Experiment 1, 16 females, mean age = 22; 25 in Experiment 2, 24 females, mean age = 22) from the University of Trento participated in the present study. At the end of the experiments, participants received a monetary compensation ranging between €6 and €12, calculated on the level of their performance during the instrumental conditioning phase. All participants had normal or corrected-to-normal vision and were unaware of the purpose of the experiment. The study was carried out in accordance with the Declaration of Helsinki and the experimental protocols used were approved by the local institutional ethics committee (Comitato Etico per la Sperimentazione con l’Essere Umano, University of Trento, Italy). All volunteers gave informed consent prior to participation.

### Stimuli, apparatus and procedure

The experiments took place in a quiet, dimly-lit psychophysics testing room. Participants rested their chin at a distance of about 60 cm from a gamma-calibrated monitor (CRT, 19”, 1024 × 768, 100 Hz) on which visual stimuli were presented. A program written using Matlab and the Psychophysics Toolbox 3.8 managed stimuli presentation, response collection and data storage.

**Figure 1:**
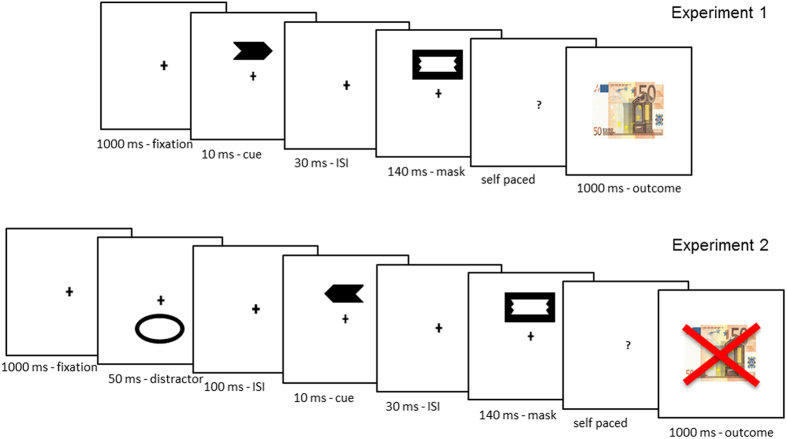
Top row. Events of Experiment 1: on each trial the cue (left or right arrow) randomly appeared either below or above the central fixation point, and was followed 30 ms later by the mask (metacontrast masking procedure). Then a central question mark appeared prompting participants to decide whether or not they were willing to take the risk of the ensuing outcome. Once participants responded by pressing one of two keys on the computer keyboard, a given outcome (monetary gain or loss) transpired depending on the type of cue (see methods for details). Bottom row. Events of Experiment 2: the sequence of events was as in Experiment 1, with the exception that, before the cue, an oval stimulus was briefly presented in the cue opposite location. The oval onset served as an attentional distractor.

**Figure 2:**
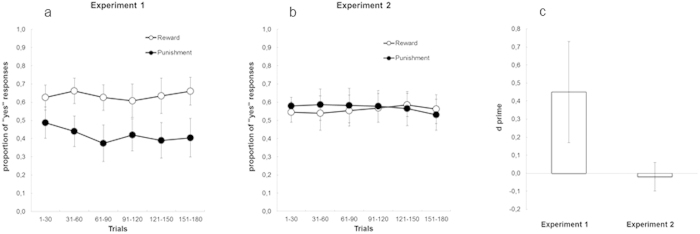
(**a**) Results of the conditioning phase in Experiment 1. Overall, the proportion of the “Yes” responses was higher for the reward-predicting cue than for the punishment-predicting cue, thus showing evidence of subliminal instrumental conditioning. (**b**) Results of the conditioning phase in Experiment 2, in which a sudden onset was used to shift spatial attention away from the cue. With this attentional manipulation, no difference emerged between the proportions of “Yes” responses to the reward-predicting cue and the punishment-predicting cue, thus showing a lack of unconscious instrumental conditioning. (**c**). The results of Experiments 1 and 2 expressed in terms of sensitivity (*d’*) of the cue-outcome association: *d’* was larger then zero in Experiment 1, but not different from zero in Experiment 2. The difference in *d’* between the two experiments was also statistically significant. Bars represent 95% confidence intervals.

**Figure 3:**
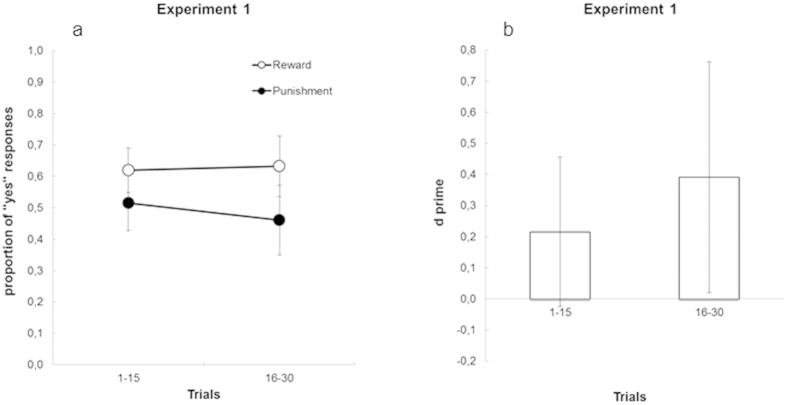
Results of the conditioning phase in the first two bins of Experiment 1. (**a**) results expressed as proportion of “Yes” responses; (**b**) results expresses in terms of sensitivity (*d’*). Learning was not significant in the first bin of trials. Bars represent 95% confidence intervals.

**Figure 4:**
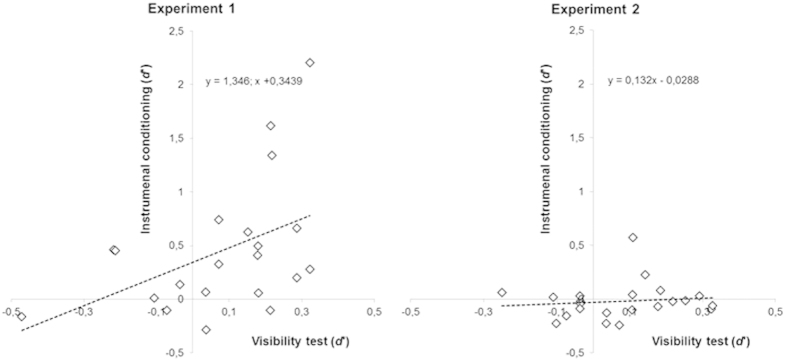
Across participants correlation between *d*’ in the subliminal instrumental conditioning and *d*’ in the visibility test, for Experiment 1 and 2. Despite in both experiments participants showed no sensitivity in the cue discrimination test, the amount of conditioning was larger in Experiment 1 than in Experiment 2, as reflected by the intercepts of the regression lines.

Left- or right-pointing arrows (1° in height, 2.4° in width) served as outcome-predicting cues, and were masked by a rectangular stimulus (1.7° in height, 3° in width) whose inner cut-out was designed to fit perfectly with the contour of both types of cues (Fig. 1, top row; see also^21^). All stimuli were displayed in black against a white background (i.e., at maximum contrast). To enhance perceptual masking, the cue and the mask appeared randomly either above or below the central fixation, at 2° eccentricity^22^.

Experiment 1 consisted of two phases (an instrumental conditioning phase, followed by a cue visibility test), and lasted less than one hour. Observers started each experimental phase at their discretion, and prior to each phase, they performed 10 practice trials.

#### Instrumental conditioning

Each participant completed 360 trials divided into four blocks of 90 trials. In 50% of the trials of each block the arrow cue pointed to the left, whilst it pointed to the right in the remaining 50%. Left- and right-cue trials were intermixed randomly. Prior to the practice trials, participants were shown a display on the computer screen depicting the two possible cue directions and positions (below and above the fixation cross). They were informed that a given cue (e.g., left arrow) was always paired with a monetary reward, while the other cue (e.g., right arrow) was always associated with a monetary loss (the cue-outcome associations were counterbalanced across the participants). However, the specific association between a given cue and the following outcome was not made explicit to participants. Participants were informed that on each trial their task was to decide whether to risk receiving a punishment in the pursuit of a monetary reward. In other terms, their goal was to maximize monetary gains, and to minimize monetary losses. It is worth pointing out that participants had to choose between “Yes, I accept the risk” and “No, I reject the risk” on the basis of their intuition, as the outcome-predicting cue was rendered consciously invisible by metacontrast masking. Participants responded by pressing one of two keys on a standard computer keyboard, without any time pressure. To increase motivation, participants were told that the amount of money they would earn in the experiment depended on the number of correct choices. The minimum they could earn was €6 (task performed at chance level, 50% correct choices), whereas the maximum was €12 (perfect task performance, 100% correct choices).

The temporal order of events in a trial is depicted in Fig. 1 (top row). Each trial started with a central fixation cross, which remained visible until a choice was requested. After 1000 ms from the onset of the fixation point, the cue appeared for 10 ms either above or below fixation, with the position randomly selected. The cue was immediately (after a 30 ms delay) replaced by the mask that remained on the screen for 140 ms. Then, a question mark prompted participants to make their choice. In case of a “Yes” response, indicating that they were willing to take the risk, there could follow one of two different visual feedbacks: an image of a €50 banknote when the subliminal cue was paired with reward, or the same banknote image marked with a red cross when the cue was paired with monetary loss. In case of a “No” response, there could follow one of two different notifications: “Sorry, you missed reward” or “Good! You avoided punishment”, depending on whether the cue was paired with reward or punishment, respectively.

#### Cue visibility test

Since we were interested in finding evidence of subliminal instrumental conditioning, cue visibility was tested for each participant at the end of the experiment. Specifically, participants performed an arrow discrimination task for 100 trials (one single block), without time pressure. The stimuli and temporal parameters of a trial were the same as in the conditioning phase, except that the masked arrows were not paired with any monetary outcomes. Left and right arrows were presented in an equal number of trials, and incorrect arrow discriminations were signaled using auditory feedback.

In Experiment 2, during the instrumental conditioning phase the cue was preceded by an outlined oval (1.8° in height, 5° in width) presented for 50 ms in the cue opposite location. The inter-stimulus-interval between the oval and the cue was set at 100 ms. The sudden oval presentation served as a distractor onset to direct attention in the cue opposite location^23^. Because we did not monitor eye movements, it could be argued that the onset distractor may have triggered a saccade toward the corresponding location, thus making the cue to be perceived more peripherally (in terms of retinotopic coordinates) with respect to Experiment 1. However, we would like the reader to note that the time between the onset of the distractor and the offset of the cue was 160 ms, a value well below the typical saccade latency in humans, which is around 200–250 ms. This makes extremely unlikely that our results were contaminated by reflexive saccades toward the distractor.

In addition, a third phase consisting in a *spatial attention task* was introduced between the instrumental conditioning and the cue visibility test, and served the purpose of assessing the efficacy of the oval onset in capturing attention. This new phase consisted of two experimental blocks of 100 trials each, during which participants performed a spatial attention task^23^. Half of the trials of each block were congruent (onset and arrow displayed in the same location), while the remaining trials were incongruent (onset and arrow displayed in different locations). Participants were instructed to respond as quickly as possible to the direction of the arrow (randomly selected from trial to trial) by pressing one of two different keys on the computer keyboard. They were also encouraged to accomplish the task with minimal errors. The temporal parameters of the oval and the arrow were the same as used during the conditioning phase, and are shown in Fig. 1 (bottom row).

## Additional Information

**How to cite this article**: Mastropasqua, T. and Turatto, M. Attention is necessary for subliminal instrumental conditioning. *Sci. Rep.* **5**, 12920; doi: 10.1038/srep12920 (2015).
